# Intravital Immunofluorescence for Visualizing the Microcirculatory and Immune Microenvironments in the Mouse Ear Dermis

**DOI:** 10.1371/journal.pone.0057135

**Published:** 2013-02-25

**Authors:** Witold W. Kilarski, Esra Güç, Jeremy C. M. Teo, S. Ryan Oliver, Amanda W. Lund, Melody A. Swartz

**Affiliations:** 1 Institute of Bioengineering and Swiss Institute of Experimental, Cancer Research (ISREC), École Polytechnique Fédérale de Lausanne, Lausanne, Switzerland; 2 Khalifa University of Science, Technology and Research, Abu Dhabi, United Arab Emirates; University of Navarra, Spain

## Abstract

Visualizing the dynamic behaviors of immune cells in living tissue has dramatically increased our understanding of how cells interact with their surroundings, contributing important insights into mechanisms of leukocyte trafficking, tumor cell invasion, and T cell education by dendritic cells, among others. Despite substantial advances with various intravital imaging techniques including two-photon microscopy and the generation of multitudes of reporter mice, there is a growing need to assess cell interactions in the context of specific extracellular matrix composition and microvascular functions, and as well, simpler and more widely accessible methods are needed to image cell behaviors in the context of living tissue physiology. Here we present an antibody-based method for intravital imaging of cell interactions with the blood, lymphatic, and the extracellular matrix compartments of the living dermis while simultaneously assessing capillary permeability and lymphatic drainage function. Using the exposed dorsal ear of the anesthetized mouse and a fluorescence stereomicroscope, such events can be imaged in the context of specific extracellular matrix proteins, or matrix-bound chemokine stores. We developed and optimized the method to minimize tissue damage to the ear, rapidly immunostain for multiple extracellular or cell surface receptors of interest, minimize immunotoxicity with pre-blocking Fcγ receptors and phototoxicity with extracellular antioxidants, and highlight the major dermal tissue structures with basement membrane markers. We demonstrate differential migration behaviors of bone marrow-derived dendritic cells, blood-circulating leukocytes, and dermal dendritic cells, with the latter entering sparse CCL21-positive areas of pre-collecting lymphatic vessels. This new method allows simultaneous imaging of cells and tissue structures, microvascular function, and extracellular microenvironment in multiple skin locations for 12 hours or more, with the flexibility of immunolabeling in addition to genetic-based fluorescent reporters.

## Introduction

The evolution of intravital imaging techniques over the last few decades has brought invaluable insight into the behavior of cells and tissue functions that govern innate and adaptive immune responses during inflammation, infection, vaccination, and cancer [1]. In live tissue, lymphatic and blood vessels have been visualized with non-invasive optical coherence tomography [2], fluorescently labeled macromolecules (e.g., dextran) [2], or near-infrared dyes like indocyanine green [4]. Cell movements can be tracked using multi-photon imaging techniques with cells expressing reporter genes [5], [6], while fibrillar matrix components can be detected by their generation of a second harmonic signal [3].

Despite these advances, there remain several unmet needs in intravital imaging. The reliance on transgenic reporter mouse models that express fluorescent proteins [4], [5] limits the range and combination of proteins that can be imaged. Furthermore, while these intracellularly expressed proteins present low phototoxicity to allow long-term imaging of the cells, they can also elicit immune responses, e.g., in transplanted GFP-expressing cells [6]. Also, since reporter protein expression is generally restricted to the intracellular compartment, it cannot be used to label extracellular structures such as basement membrane (BM) proteins or tissue deposits of chemokines or growth factors. Other frequently used labeling approaches include the use of fluorophores such as rhodamine 6G or acridine red [7], [8] which, upon i.v. injection, accumulates in leukocytes, or live cell dyes can be applied *ex vivo* to specific cell types that are then re-introduced into the tissue [9], [10]. However, the lack of specificity, potential adverse effects on cell metabolism, and high phototoxicity that cannot be controlled with extracellular administration of antioxidants are major drawbacks that potentially preclude the use of these probes for prolonged live imaging [1], [9]–[11]. Indirect labeling with antibodies against extracellular or cell surface antigens offers flexible cell-type and matrix-specific detection [12]–[14], but this approach can result in immunotoxicity mediated by labeling antigen-antibody immunocomplexes that trigger complement activation and phagocytosis. Furthermore, interference with a target antigen may affect its biological function [7]. *Ex vivo*, live cells have been overlaid onto excised and immunostained tissue preparations, for example to While this approach reduces the problems of complement and leukocytes-dependent immune toxicity, the excised skin lack the physiological context of living tissue, where the dynamic interactions between blood, interstitial, and lymphatic fluid flows affect extracellular gradients of chemokines and growth factors that are important for guiding the migration of e.g. tumor cells and dendritic cells (DCs) [17]–[19].

Here we describe an intravital immunofluorescence (IF) method for epifluorescence stereomicroscopy that uses the surgically exposed and immunolabeled living ear dermis. Importantly, fluid and cell transport functions can be simultaneously studied in the context of various extracellular structures and chemokine deposits at the single cell resolution. Immunotoxicity of antigen-antibody labeling is avoided by specific blocking of the leukocyte Fcg receptors and by exhausting complement in the fluid phase with irrelevant immunocomplexed mouse polyclonal IgGs. After immunostaining, the amplified fluorescence is protected from photobleaching with ascorbate buffer, allowing long-term imaging (12 hours or more) of cell migration in normal or drug-challenged tissues that are supported by functional blood and lymphatic vessels, while simultaneously protecting cells from harmful phototoxicity. The surgical exposure of the ear dermis is made possible by the fact that the mouse ear is composed of two skin layers that are independently innervated and fed by separate blood and lymphatic circulations. As assessed by fluid and cell extravasation measurements, the surgery resulted in minimal vessel leakage and leukocyte extravasation compared to that induced by VEGF and anaphylatoxins, respectively.

The thinness (∼100 mm) of the separated ear dorsal skin allows conventional epifluorescence stereomicroscopy to be used, which has multiple advantages as compared to two-photon or confocal imaging. These advantages include an adjustable depth of field, increased range in magnification, higher imaging speed that allows multiple locations to be simultaneously imaged and a much lower cost and degree of expertise required. Its main disadvantage over confocal (two-photon) imaging is the lack of ability to optically section in the z-direction, which can be important for verifying intravasation. On the other hand, the intravital IF method described here can also be used with two-photon, or, due to the thinness of the ear tissue, with confocal systems if desired. The method can also be used in combination with fluorescent reporter probes.

We demonstrate a variety of uses of intravital IF, including assessment of microvascular permeability and lymphatic drainage, immunolabeling of extracellular matrix and tissue components, and several examples of leukocyte migration and extravasation using both genetic probes (leukocytes from EGFP reporter mice transfused i.v. and chimeras of EGFP-bone marrow, stroma wild-type) as well as antibody labeling (e.g., CD45, CD11c, CD11b, and MHCII). The power of combining these probes is demonstrated by data showing dermal dendritic cells (DDCs) and bone marrow dendritic cells (BMDCs) patrolling the interstitial space and migrating into lymphatic vessels. We also show transfused leukocytes extravasating blood vessels and migrating to the regions of concentrated extracellular chemokine stores of the lymphoid chemokine CCL21. Thus, intravital IF offers broad utility in observing physiological and pathological processes in the skin in the context of a functioning blood and lymphatic vasculature.

## Methods

### Ethics Statement

This study was carried out in strict accordance with the Swiss Animal Protection Act, the ordinance on animal protection and the ordinance on animal experimentation. We confirm that our Institutional Animal Care and Use Committee (IACUC), named Commission de Surveillance de l’Etat de Vaud (Permit Number: 2316 and 2464), specifically approved this study. Human serum was obtained from healthy volunteers, under which approval was obtained from the Commission Cantonale d’Ethique de la Recherche sur l’Etre Humain. Participants provided their written informed consent to participate in this study. To document this process, the official form from EPFL, the hosting institution, was filled each time the blood was drawn.

### Ear Preparation

Unless otherwise noted, BALB/c mice were used in all experiments between ages 8–12 weeks, and were purchased from Charles River (Orleans, France). Three days before the experiment, the mouse head was shaved and the ears were briefly depilated (10–15 seconds) and rinsed with water [20]. Mice were anesthetized with 2.5% isofluorane (Minrad Inc., Buffalo, NY) using a humidified delivery system (Rothhacher GmbH, Berne, Switzerland), and thereafter maintained under 1.5% of isofluorane for the duration of the experiment. The preparation is detailed in Suppl. Figure 1 and Video S1. Briefly, the ventral skin of the ear was cut along the antihelix of the mouse pinna, followed by ligation and separation of cartilage and muscle using the blunt edge of a scalpel. Two additional 1-mm cuts were made at the edge of the ear to prime the separation of the ventral and dorsal parts. The ventral skin and cartilage were then gently pulled apart with curved forceps to separate the ventral and dorsal skin, exposing the dermis at the posterior and midsections of the ear and the hypodermis at the anterior side.

**Figure 1:**
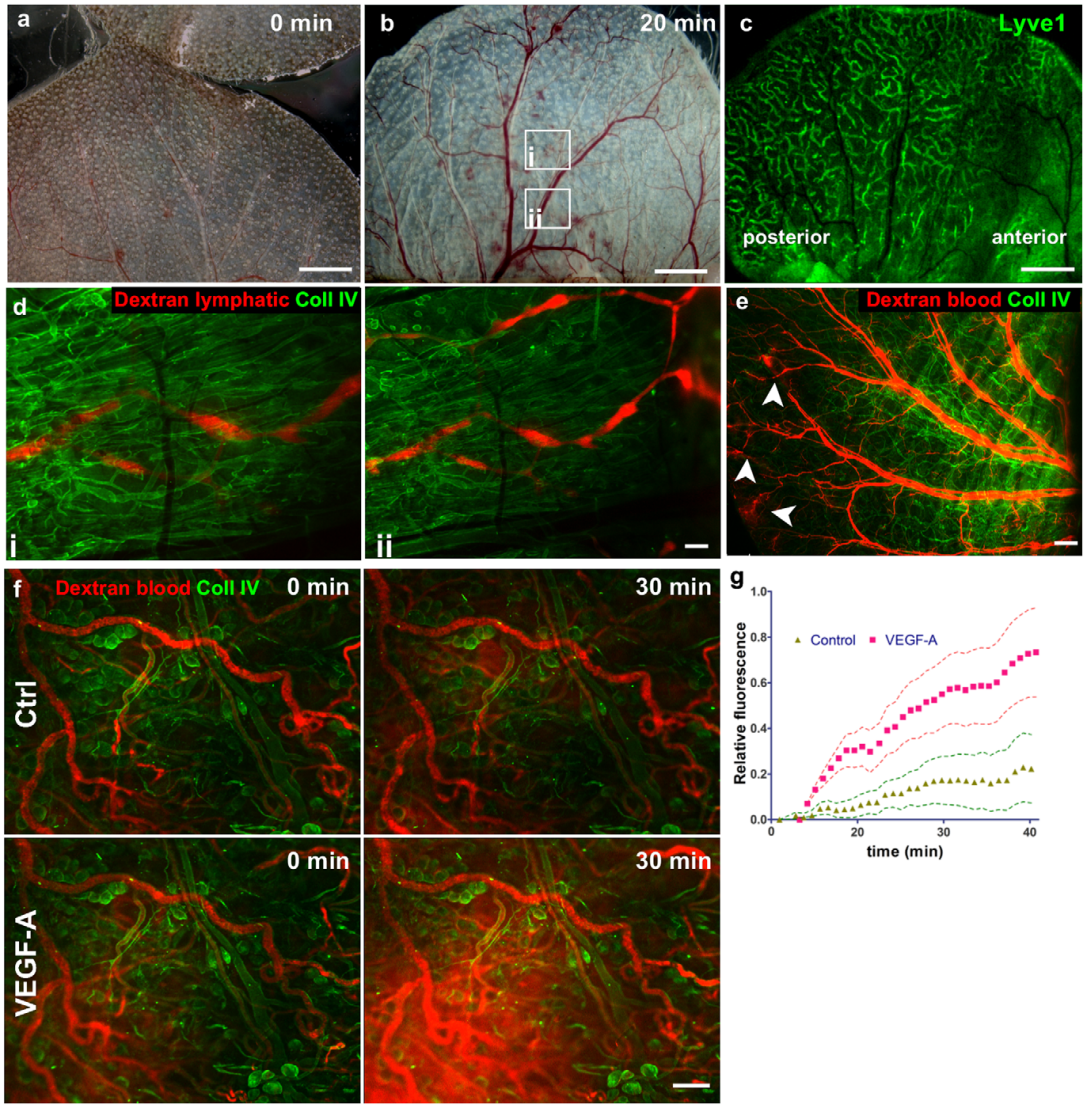
Blood circulation in the surgically exposed dorsal ear dermis is functional. (**a, b**) Bright-field images showing the blood circulation (**a**) immediately after surgery and (**b**) after 20 min where circulation appears fully restored in all major blood vessels. (**c**) Lyve1 staining shows the lymphatic vessel network, where the anterior part of the ear cannot be imaged with wide-field microscopy due to underlying adipose tissue of the hypodermis. (**d**) Functional drainage of TRITC-dextran by lymphatic vessels. TRITC-dextran was injected intradermally in the top of the dorsal skin that was stained for collagen IV. (**e**) Functionality of blood vessels in the exposed dermis as visualized with intravascular TRITC-dextran (red) showing damaged vessels (arrowheads) that leak dextran into the interstitial space. (**f**) Blood vessel permeability was assessed in the collagen IV-stained ear tissue after i.v. injection of 155 kDa TRITC-dextran. (**f**) Basal level of leakage over 30 min (Ctrl-control; top) and in the same tissue after addition of 1 µg/ml VEGF-A (bottom). (**g**) Relative increase in fluorescence intensity in the extravascular space as a function of time, showing quantification of vascular leakage. Triangles and squares represent average values and dotted lines, their standard errors from 10 imaging fields. p<0.0001 of the difference between the two lines. The following images come from the same experiment (separated by semi-colon): Fig. 1a and 1b; Fig. 1c; Fig. 1d; Fig. 1e; Fig. 1f. Scale bars in a, b, and c, 2 mm; d, f, 100 µm; e, 500 µm.

For experiments involving a topical application of CCL21, a fragment of dorsal skin was removed and the dermis was incubated with 50 ng/ml or 1 ng/ml CCL21 in aprotinin-Ringer’s buffer for 15 min followed by rinsing in Ringer’s buffer and subsequent removal of the residual dorsal dermis. For determining lymphatic drainage function, half of the ventral skin was removed, and the exposed middle part of the ear was stained for collagen IV. Then, 1 µl of TRITC-dextran 125 kDa (10 mg/ml) was injected intradermally and the draining area of the dermis was imaged immediately afterwards for 30 min.

### Intravital Imaging Setup

Surgical procedures and live imaging were performed using a fluorescence stereomicroscope with a motorized stage (M250 FA, Leica Microsystems CMS GmbH, Wetzlar, Germany) equipped with a 1× lens (linear system magnification from 7.5× to 160×) or 2× lens (linear system magnification from 15.6× to 320×), with a resolution range between 7.89 µm (at 15.6× magnification) and 0.95 µm (at 320× magnification), respectively. Images were collected using a DFC 350 FX camera controlled by LAS AF software (both from Leica). Mice were maintained under 1.5% isofluorane and their temperature was monitored and controlled throughout the experiment (DC Temperature Control System, FHC Inc., Bowdoin, MA).

### Intravital Immunofluorescence Staining

The exposed dorsal ear dermis was placed on a sheet of Parafilm atop a 8-mm block composed of 8 glued microscope slides to support the mouse ear. All antibodies were mixed in Ringer’s buffer (102 mM NaCl, 5 mM KCl, 2 mM CaCl2, 28 mM sodium lactate) supplemented with 125 IU/ml (2.5 mg/ml) aprotinin (herein referred to as “aprotinin-Ringer”) and kept at 4°C before being pipetted onto the exposed ear dermis. Fcγ-blocking buffer was prepared by mixing 10 µl human serum with 2 µl mouse serum raised against human IgG in 100 µl aprotinin-Ringer. The blocking solution was applied on the skin for 15 min before and during the staining with antibodies. Aprotinin served to inhibit initial bleeding from injured capillaries by decreasing anti-thrombolytic conditions. 100 µl of the blocking solution was then applied on the exposed skin for 15 min before applying blocking solution with labeling antibody. Then, primary antibodies (10 g/ml) targeted anti-cell surface markers were applied for 15 min in blocking solution, and after 2 brief rinses with approximately 5 ml of Ringer’s buffer, appropriate secondary antibodies or streptavidin conjugates with Pacific Blue, Alexa-488, Alexa-594 or Alexa-647 (10 µg/ml) were added to the tissue for another 15 min. After two additional rinses, the ear was immobilized onto the glass slide by applying 0.5 µl of surgical glue to each end of the intact skin of the eminentia conchae at the ear base and gently flattening the dorsal ear dermis onto the glass. The exposed dermis was then incubated with ascorbate-modified Ringer’s (herein referred to as “ascorbate-Ringer”, containing 140 mM sodium ascorbate, 25 mM HEPES, 4 mM KCl and 2 mM CaCl2, at a pH of 7.5). Where indicated, tissue was stimulated with 20% freshly isolated mouse serum activated with 0.2 IU/ml cobra venom factor (which activates the complement cascade) or 1 µg/ml bacterial lipopolysaccharide (LPS) in ascorbate-Ringer. For long-term imaging, the outlet of the needle (connected to a reservoir containing ascorbate-Ringer) was mounted under the coverslip 0.5 cm from the ear and ascorbate-Ringer was constantly delivered to the chamber under the coverslip by peristaltic pump at 1 µl/min. The ear and the 30G needle were covered with a coverslip, and 10 to 20 pre-defined fields were imaged.

### Reagents

The following reagents were used in the study: isofluorane (Minrad Inc., Buffalo, NY), Ringer’s lactate buffer (B. Braun Medical AG, Sempach, Germany), Dulbecco’s modified Eagle’s medium (DMEM, Gibco Invitrogen, Grand Island, NY), fetal bovine serum (FBS, Gibco Invitrogen), penicillin-streptomycin-amphotericin B (Gibco Invitrogen), trypsin (Gibco Invitrogen), ultra-pure bacterial lipopolysaccharide (LPS, InVivogen, San Diego, CA), cobra venom factor (Quidel, San Diego, CA), Texas Red–dextran 155 kDa (Invitrogen, Carlsbad, CA), CMTPX CellTracker (Invitrogen), aprotinin (Elastin, Owensville, MO), sodium ascorbate (Sigma). The following anti-mouse antibodies were used: biotinylated rabbit anti-Collagen type IV (Abcam), rabbit anti-Lyve1 (RELIATech, Wolfenbüttel, Germany), biotinylated rat-anti perlecan (Abcam), hamster anti-CD31 (2H8, GeneTex, San Antonio, TX), rat anti-CD45-biotinylated (30-F11, BD Bioscience, Franklin Lakes, NJ), rat anti-CD11b (M1/70, Ebioscience, San Diego, CA), hamster anti-CD11c (HL3, BD Bioscience), rat anti-MHCII (I-A/I-E, BD Bioscience), rat anti-VE-cadherin (11D4.1, BD Bioscience), mouse anti-α smooth muscle actin (1A4, Abcam). Secondary detection reagents were all from Invitrogen: streptavidin-Pacific Blue, streptavidin Alexa 647, anti-rat Alexa 594, anti-rat Alexa 594, anti-hamster Alexa 594, and anti-rabbit Alexa 488. In some cases, a fluorescently tagged anti-Lyve-1 antibody was used; labeling was accomplished by mixing 0.5 mg/mL Lyve-1 antibody with 1 mg/mL AlexaFluor 488 N-hydroxy-succinimidyl-ester (Invitrogen) in PBS (pH 8) for 1 h at room temperature followed by purification in a desalting column and verification of labeling by absorbance of the purified conjugate at 280 and 494 nm. Human serum was obtained from healthy volunteers.

### Whole-mount Staining and Confocal Imaging

Mice were sacrificed with CO_2_ and exsanguinated by intracardiac perfusion with 20 ml Ringer’s buffer (supplemented with 10,000 IU/L heparin, 0.1% glucose, 0.1% procaine and 25 mM HEPES; pH 7.5, 330 mOsM) at a constant gravitational pressure of 120 mm Hg. Ringer’s buffer was then changed to osmolarity-corrected zinc fixative (Zn fix) solution [21] (4.5 mM CaCl_2_, 52 mM ZnCl_2_, 32 mM Zn(CF_3_COO)_2_, 2 mM Tris, 38 mM glycine; pH 6.5, 340 mOsm/L) and ears were cut and placed in ice-cold Zn fix with 1% Triton x-100 for at least 24 h. Afterwards, the ventral part of the skin was removed together with cartilage and muscles, and the dorsal dermis was washed in TBS (140 mM NaCl, 25 mM Tris, pH 7.5) for 6 h, blocked with 0.5% casein in TBS (blocking solution) for 2 h, and incubated with anti-VE-cadherin, anti-Lyve-1, and biotinylated anti-collagen type IV antibodies (10 µg/ml) in blocking buffer for 24 h. After washing in TBS with 0.1% Tween 20, tissue was incubated with the appropriate secondary antibody (10 µg/ml) for another 24 h, washed in TBS and dehydrated with 70% and 100% ethanol before it was cleared with 2∶1 benzyl benzoate/benzyl alcohol solution (refractive index 1.56) supplemented with 25 mg/ml propyl gallate (antioxidant). The tissue was mounted on a glass slide and imaged using a confocal microscope (Leica SP5 with 20× or 63× lenses, NA 1.40). Image stacks were analyzed using Imaris 7.1 (Bitplane AG, Zürich, Switzerland).

### Blood Transfusion

EGFP-expressing mice (CByJ.B6-Tg(UBC-GFP)30Scha/J) were sacrificed by cervical dislocation and 500 µl blood was immediately harvested by intracardiac puncture into a tube containing 2 mg of EDTA. Blood cells were washed 3 times in 15 ml Ringer’s buffer, resuspended in Ringer’s buffer to the drawn blood original volume of 500 µl, and injected into tail veins of the recipient mice, whose ears had been earlier immunostained for Lyve1 or collagen type IV.

### GFP Chimeric Mice

Recipient 8-week old BALB/c mice received 800 rad in two equal doses, 3 hours apart. Treated mice were reconstituted with 5×10^6^ syngeneic bone marrow cells isolated from an EGFP mouse [22]. Drinking water was supplemented with 2 mg/mL Dafalgan, 800 µg/mL sulfamethoxazole, and 160 µg/mL trimethoprim (Bactrim), and wet food was given. Two months later, the level of chimerism in blood was tested by flow cytometry and mice with >95% EGFP-expressing CD45^+^ cells were used for the live imaging experiments.

### Cell Isolation and Culture

#### Peritoneal macrophages

Mice were killed by cervical dislocation and unstimulated peritoneal macrophages were collected by injecting and withdrawing 10 mL harvest medium with a 19 G needle. Collected cells were plated into Petri dishes and stimulated overnight with 1 µg/ml LPS. After 24 h, cells were harvested with PBS and labeled with rat anti-CD11b antibody for 15 min at 4°C. After washing, cells were incubated for 15 min with Alexa 594-labelled goat anti-rat IgG, washed again, and pipetted onto the pre-stained ear. 30 min later, unattached cells were washed away with ascorbate-Ringer and the ear was imaged as described above.

#### Bone marrow-derived dendritic cells (BMDCs)

BMDCs were prepared as described [23]. Briefly, a EGFP-expressing mouse (CByJ.B6-Tg(UBC-GFP)30Scha/J) was killed by cervical dislocation and the femur and tibia bone marrow cavities were washed with harvest medium. Cells were seeded on cell culture plates in MEM supplemented with 20% FBS and 20 µg/ml GM-CSF and cultured for 7 days. At that time, 1 µg/ml LPS was added to the culture medium, and after 24 h, cells were harvested with PBS/5 mM EDTA. 10^5^ BMDCs in 100 µl culture medium were overlaid onto the pre-stained ear, and after 30 min, unattached cells were washed away with ascorbate-Ringer and the ear was imaged as described.

#### Analysis of leukocyte migration

The dynamics of leukocyte migration were analyzed at the population level, comparing migration towards a vessel area that stained positively for CCL21+ to that toward a vessel devoid of CCL21 staining. First, regions of interest (ROIs) were constructed around positive or negative vessels, and leukocyte influx was analyzed at different timepoints using a cross-correlation analysis against the first image (time = 0) using a custom MATLAB (Mathworks, MA, USA) code. In this way, the relative leukocyte influx represents the change in leukocyte density within the ROI compared to the initial image; 0 represents no change (and thus no influx), while 100% refers to the maximum (e.g., leukocytes fill the entire image). Regions of interests were selected to compare lymphatic collecting vessels without visible CCL21 staining (control) with those containing CCL21.

### Statistical Analysis

Cell migration data was analyzed with Prism (GraphPad, San Diego, CA). Data sets were evaluated using an unpaired t-test with Welch’s correction, and considered significant when P≤0.05. Slopes were compared by analysis of covariance and considered significant when P≤0.05.

## Results

### Functional Blood and Lymphatic Circulation of Surgically Exposed Ear Dermis

The procedure for removing the ventral portion of ear skin and cartilage and exposing the dorsal hypodermis-free dermis is shown in Figure S1 and Video S1. Since the two skin flaps are fed by independent vascular beds, the procedure appeared to cause very minimal tissue injury with any initial bleeding limited to capillaries of the dorsal ear skin (Figure 1a and Video S2 and S3). Within 10–20 min, blood perfusion was fully restored after the ear handling during the surgery (Figure 1b). Also, since the fat-rich hypodermis expands from the anterior side towards the middle part of the ear, covering visual access to blood and lymphatic vessels, only the posterior part of the skin could be used for wide-field epifluorescence imaging of the blood and lymphatic circulation (Figure 1c). Lymphatic vessels remained intact and functional, as shown by the accumulation of fluorescently labeled dextran in collecting lymphatic vessels after a dextran solution was injected intradermally in the top of the ear (Figure 1d) or after the dextran solution had been injected i.v., had leaked into the interstitial space and was collected into a lymphatic vessel (Video S4). Because the exposed dermis was facing atmospheric oxygen pressure during the reperfusion, we did not expect hypoxia to significantly affect the tissue in first minutes after the surgery. Furthermore, the separation of dorsal and ventral skin fragments did not appear to affect perfusion and functionality of major blood vessels (Figure 1e). Since any hemorrhaging resulting from skin separation was resolved within minutes after the surgery, local tissue injury could be detected by TRITC-dextran leakage from damaged blood vessels (Figure 1e). Blood vessel leakage was considered as a marker for surgery-related tissue stress and it was measured in various post-surgery conditions (Figure 1f, g). This method is analogous to a standard Miles permeability assay where extravascular accumulation of a labeled macromolecule is assessed [3], [24]. One hour after the surgery, TRITC-dextran (155 kDa) was injected intravenously and images were recorded for 30 min. The observed basal leakage was minimal compared to that induced by a topical application of 20 µl of 1 µg/ml VEGF-A (Figure 1g), indicating that even though the surgically exposed dermis had not fully recovered to its steady state, the addition of VEGF could further stimulate blood vessel leakage.

Respiration-dependent drift of the XY-imaged fields was reduced by fixing each side of the intact skin of the ear eminentia conchae to the supporting glass slides with 0.5 µl of the surgical glue, which did not obstruct the tissue or affect blood flow. To prevent tissue drying, as well as to stabilize the focal distance, a glass coverslip was placed on the ear, glued to the supporting slide base, and perfused underneath with ascorbate-Ringer’s solution (Figure S1g). The coverslip protected the ear from drying and also mimicked the presence of avascular cartilage, providing similar oxygenation conditions as in intact ear.

### Protection against Immuno- and Photo-toxicity

**Figure 2:**
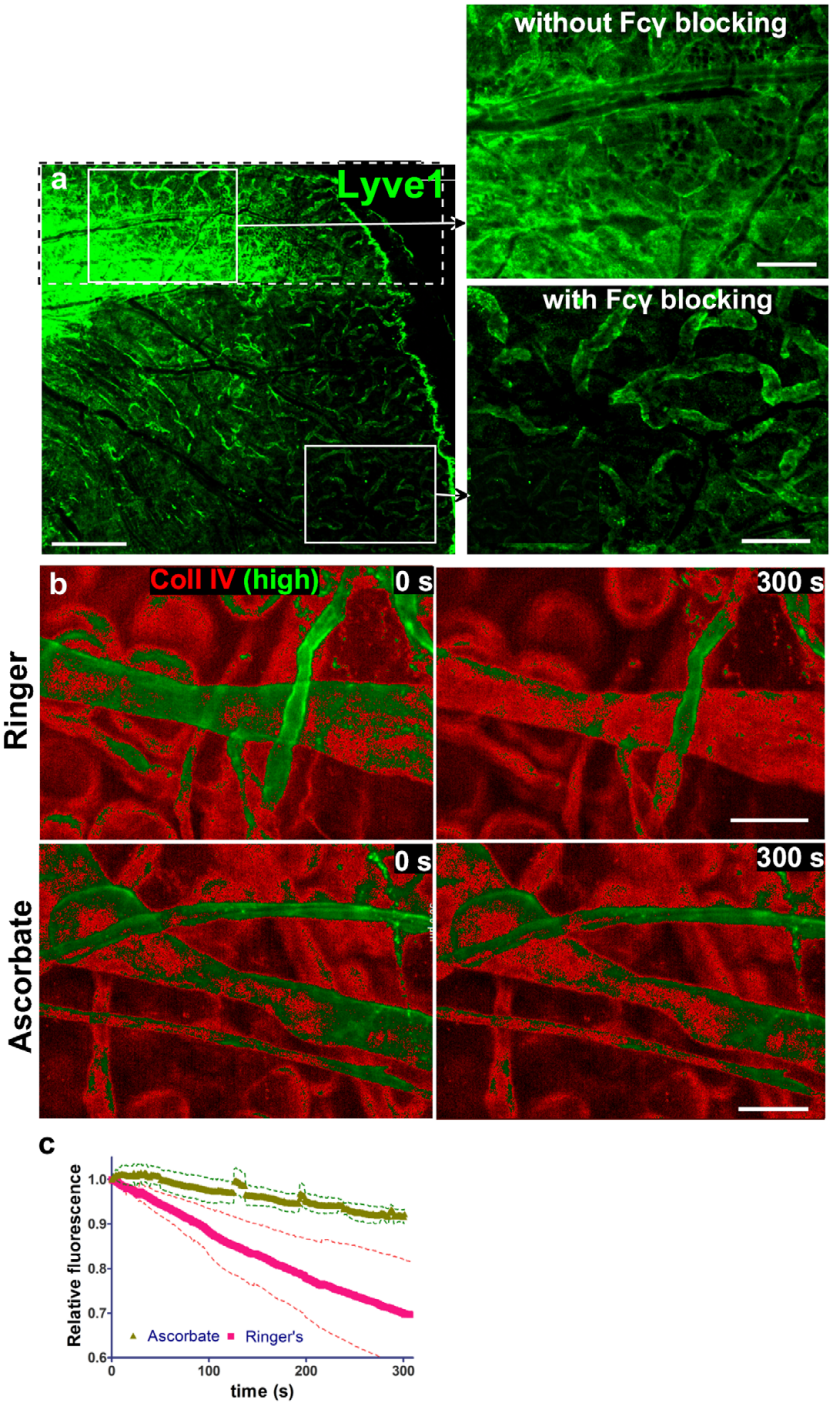
Intravital immunostaining and live imaging of dermis is possible after F_c_γ receptor blocking and inhibition of phototoxicity. (**a**) A small posterior region of the ear was first surgically exposed (dotted box), stained for Lyve1 and detected with a secondary antibody. Subsequently, the entire ear was exposed, blocked with irrelevant immunocomplexes for 15 min, and probed for Lyve1 as described. Top inset: without Fcγ receptor blocking, high background results from immunocomplexes that form between primary and secondary antibodies and bind Fcγ receptors on tissue-resident immune cells. Bottom inset: this background is largely gone with pre-blocking of Fcγ receptors with irrelevant pre-formed immune complexes. (**b**) Photobleaching was minimized by replacing sodium chloride with sodium ascorbate in Ringer’s buffer that bathed the ear tissue during imaging. Tissue was stained with a biotinylated antibody against collagen IV and streptavidin-Alexa 647 (red), and then constantly imaged for 300 s in either unmodified Ringer’s buffer (upper panel) or ascorbate-Ringer (lower panel); to demonstrate the extent of photobleaching. The brightest 25% of pixel intensity values are shown in green (“high”). Note that the protecting effect of the ascorbate can be underestimated as the single exposure time for Ringer (control) was 227 ms while the exposure time for ascorbate was 427 seconds. (**c**) Quantification of fluorescence decay over time (30% in Ringer’s vs. 5% in ascorbate-Ringer), normalized to the initial fluorescence, averaged from 3 imaging fields. Standard errors are shown by the dotted lines and the difference probability between the two lines was p<0.0001. Scale bars in a, 200 µm b, 50 µm.

Immunotoxicity of the tissue-resident immune cells, mediated by their Fcγ receptors, was blocked by pre-incubating tissue with irrelevant, non-fluorescent mouse polyclonal IgGs raised against and immunocomplexed with human IgGs (Figure 2a). This step protected cells and extracellular structures, labeled *in vivo* with primary and secondary antibodies, from humoral-dependent immune clearance by saturating the Fcγ receptors as well as protected labeled cells from potential complement-mediated attack [25]. This Fcγ blocking step also decreased background fluorescence due to competitive binding to Fcγ receptors (Figure 2a). Note that this is not equivalent to blocking of the nonspecific binding sites with serum, which is normally used in immunohistology, since normal serum does not contain immunocomplexes that can block Fcγ receptors. Alternatively to indirect immunofluorescence, the use of fluorescently labeled primary antibodies would reduce the effects of Fcγ-dependent toxicity; however, this resulted in a lower signal-to-noise ratio as compared to using a labeled secondary antibody that amplifies the signal. For example, a fluorescently labeled anti-Lyve1 antibody produced a five-fold weaker signal with higher background autofluorescence than that produced by indirect labeling (Figure S2a).

In contrast to whole-mount staining of fixed ear skin, which requires overnight incubation for each antibody application step and extensive (4–6 h) washing [26], intravital IF required only 15 min incubation with very brief washing (Video S5). This rapid immunostaining was dependent on the interstitial flow and drainage into functional lymphatic vessels, as demonstrated after disrupting lymphatic vessels in a portion of the ear (Figure S2b). Because the direction of fluid convection (i.e., from perfused capillaries to lymphatic vessels) concentrated the antibodies at lymphatic vessels, these structures stained more strongly than functional blood vessels for the pan-endothelial junction molecule-1 (PECAM-1, CD31) (Figure S3a). In contrast, in fixed tissue blood vessels are more efficiently labeled with anti-PECAM-1 staining (data not shown), where interstitial flow directed towards lymphatic vessels is absent. These results suggested that lymphatic drainage provided intrinsic clearance of the applied antibodies, leading to very rapid staining.

The relaxation of a light-activated fluorophore results in either fluorescence emission or bimolecular reactions that produce reactive oxygen species (ROS) [27], which can cause both fluorophore photobleaching as well as cytotoxic effects including arteriole vasospasm and leakage, leukocyte adhesion to endothelium, and thrombus formation [28]. In addition, extensive ROS formation can lead to tissue hypoxia [29]. Only in the case of extracellular staining of live tissue, ROS-induced toxicity and fluorophore photobleaching can be prevented with application of antioxidants such as vitamin C (ascorbate), and thus we modified Ringer’s solution by replacing 140 mM NaCl with the same molarity of sodium ascorbate. This efficiently protected the fluorescence from photobleaching and the cells from phototoxicity-induced cell death (Figure 2b–c). This sodium ascorbate protection lasted throughout the duration of our experiments, at least 12 h of imaging with no adverse effect on cell motility (Video S6), suggesting that adequate levels of blood-delivered nutrients and oxygen, critical for leukocyte migration [12], were maintained during imaging.

### Intravital Imaging of Tissue Structures

**Figure 3:**
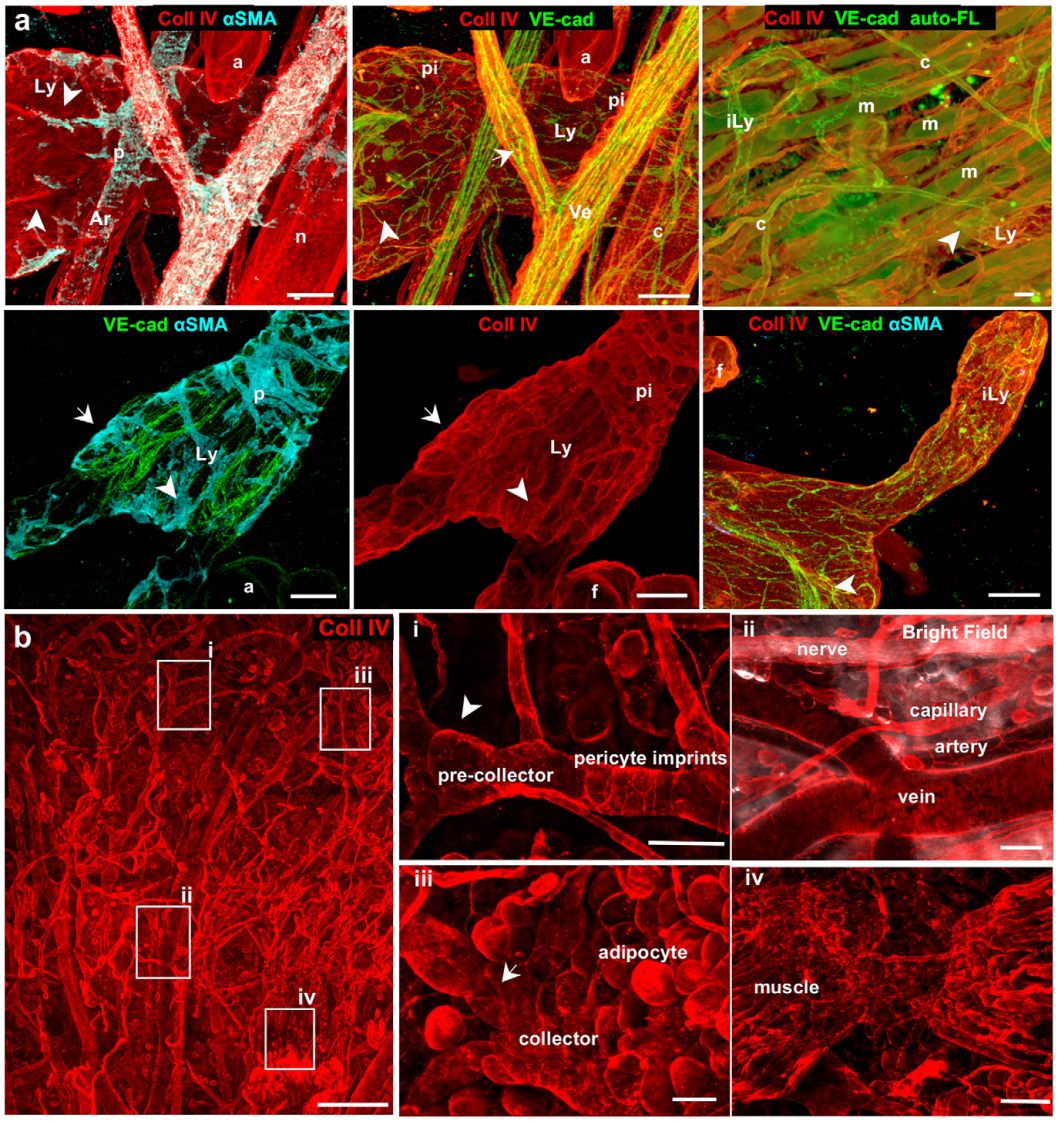
Antibodies against collagen IV (basement membrane) served to identify most tissue structures in the dermis during intravital imaging. (**a**) Confocal imaging of the whole-mount stained tissue for the markers indicated. (**b**) Intravital immunofluorescence (IF) on the dorsal ear using collagen IV reveals the same structures except arterial smooth muscle cells (SMCs) and most capillary pericytes, with insets (i)–(iv) corresponding to zoomed areas shown at left by indicated boxes. Lymphatic collectors (Ly), initial lymphatics (iLy), veins (Ve), arteries (Ar), capillaries (c), nerve fibers (n), adipocytes (f), muscle fibers (m), SMC (p), collagen impressions of SMC (pi). Arrows indicate bicuspid valves of veins and collecting lymphatics; arrowheads indicate asymmetric valves of the pre-collecting lymphatics. Detection reagents: (a), collagen IV-streptavidin Alexa 700, VE-cadherin-Alexa 488, and αSMA-Alexa 594; (b), collagen IV-streptavidin Alexa 647. Scale bars in a, 20 µm; b, 500 µm (left) and 50 µm (insets).

While it would be advantageous to visualize tissue structures like blood and lymphatic vessels, adipocytes, nerves, and muscle in addition to the markers of interest, immunostaining each of these structures specifically is limited by the selection of fluorescent labels and filter sets available. Instead, one can label all of these structures simultaneously by immunostaining for laminin (not shown), collagen type IV or perlecan, the major components of basement membrane (BM), and readily differentiate between the structures by their morphological hallmarks (Figure 3).

To demonstrate the usefulness of collagen IV staining for tissue microenvironment visualization, we compared the staining patterns of collagen IV in fixed dermis using a confocal microscope (Figure 3a) with those of α-smooth muscle actin (αSMA) and VE-cadherin, which allowed veins and large collecting lymphatic vessels to be readily distinguished by their sparse smooth muscle cell (SMC) coverage, bicuspid valves in veins and collecting lymphatics, and asymmetric valves in pre-collecting lymphatic vessels [30] (Figure 3). In both vessel types, collagen IV staining also revealed the imprints of SMCs and lymphatic valves, while nerve fibers and adipocytes were devoid of such impressions. Thin and straight arteries had dense BM, while torturous capillaries were associated with αSMA-negative pericytes. Autofluorescence of striated muscle fibers typically seen in whole-mount staining was not detectable with intravital IF, where muscle fibers could be distinguished from other collagen IV^+^ tubular structures by their densely packed arrangement. The BM around blind-ended and pericyte-free initial lymphatics was less extensive than that around collecting vessels, but still detectable in both fixed tissue as well as in intravital IF (Figure 3a and Figure S3b). Lyve1^+^ initial lymphatics were also distinguished from SMC-covered podoplanin^+^ pre-collectors (Figure S3c) located in deeper focal plane (Figure S3d).

### Detection of Matrix-bound Extracellular Pools of CCL21 Chemokine

**Figure 4:**
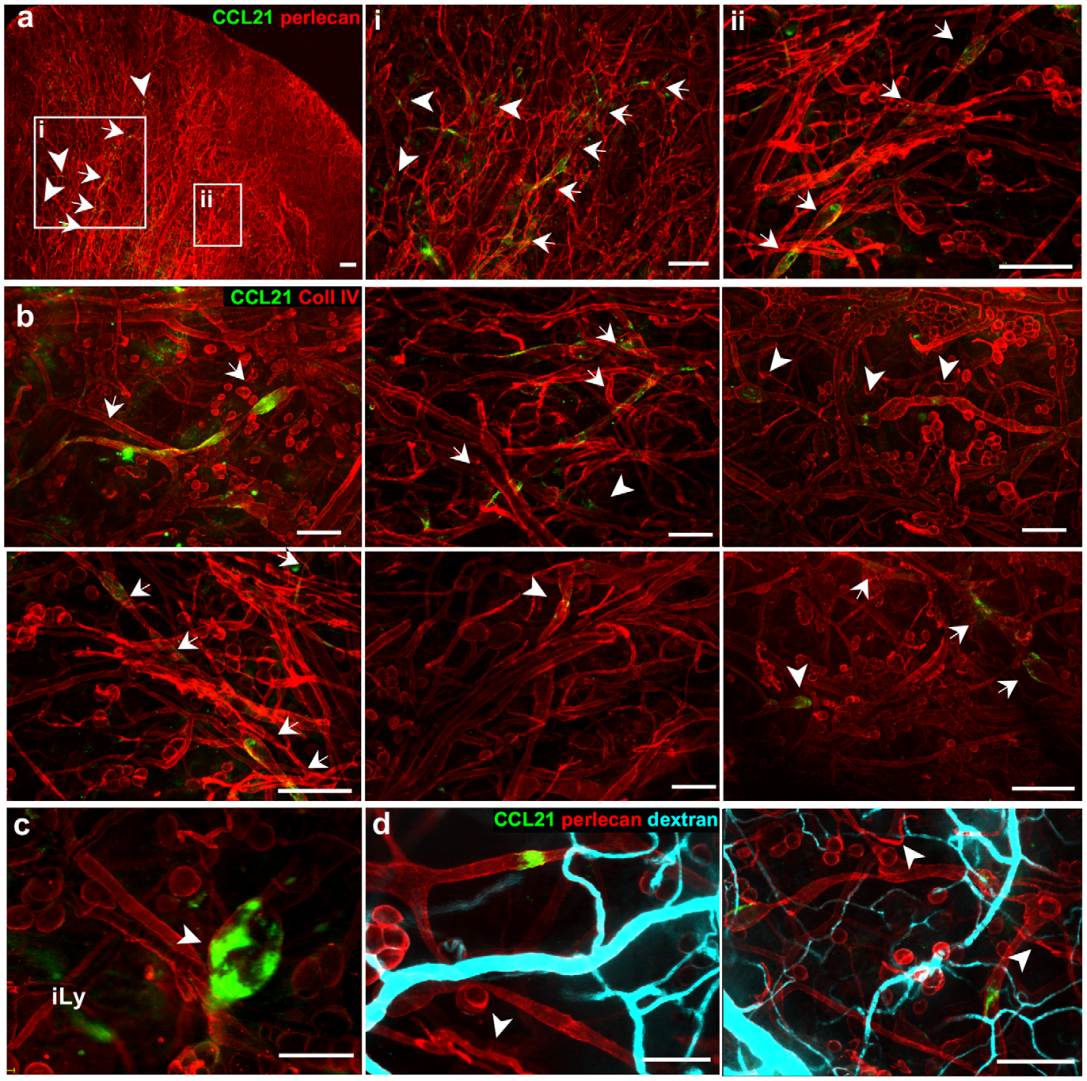
Live immunostaining detects only extracellular basement membrane-bound CCL21, while staining on fixed tissue reveals intracellular as well as extracellular CCL21. (**a–b**) In the live ear dermis CCL21 accumulated in patches (arrowheads) and along continuous lymphatic segments (arrows), and co-localized with (**a**) perlecan and (**b**) collagen IV, both basement membrane components of the collecting lymphatic vessels. (**c**) Occasionally observed CCL21-positive (green) initial lymphatic vessels (iLy) stained more weakly for collagen IV (red). (**d**) Extracellular CCL21 deposits (green) were seen around lymphatic vessels in the exposed ear skin did not correlate with injured blood vessel, as determined by i.v. injection of TRITC-dextran (cyan) that leaked from areas of injured vessels (arrowheads). The tissue was pre-stained for perlecan and CCL21 before dextran injection. Scale bars in a, b and d (left), 400 µm; c and d (right), 100 µm.

The chemokine CCL21, a ligand for CCR7 that binds strongly to basement membrane heparan sulfate proteoglycans and that is expressed by lymphatic vessels in skin, is critical for the homing of CCR7^+^ dendritic cells and naïve T cells into lymphatics and to the lymph node [17]. In fixed tissues, immunostaining labels both intracellular and extracellular CCL21, which appears in lymphatic endothelial cells and along their basement membrane [13], . In contrast, intravital IF reveals only the extracellular deposits of CCL21, which were found in discrete and sparse patches (Figure 4a–c), mostly in collecting lymphatic vessels and only rarely on initial lymphatics. In 10 ears stained for CCL21, each had showen similar staining patterns of extracellular CCL21 deposits limited to patches and discontinuous lymphatic segments along the lymphatic collectors. On average, a single dorsal ear contained only 1–3 lymphatic collector segments that were strongly positive for CCL21. Quantitatively, only 17±14% (SD) of the surface of lymphatic collectors were associated with CCL21. Very few initial lymphatics (lymphatic capillaries) were found to stain for CCL21, which is expected since initial lymphatics have much less basement membrane [15] and thus less capacity for binding CCL21 (Figure 4c). Finally, we found no correlation between the locations of CCL21 patches with areas of tissue injury, as seen by blood vessel perfusion (Figure 4d).

**Figure 5:**
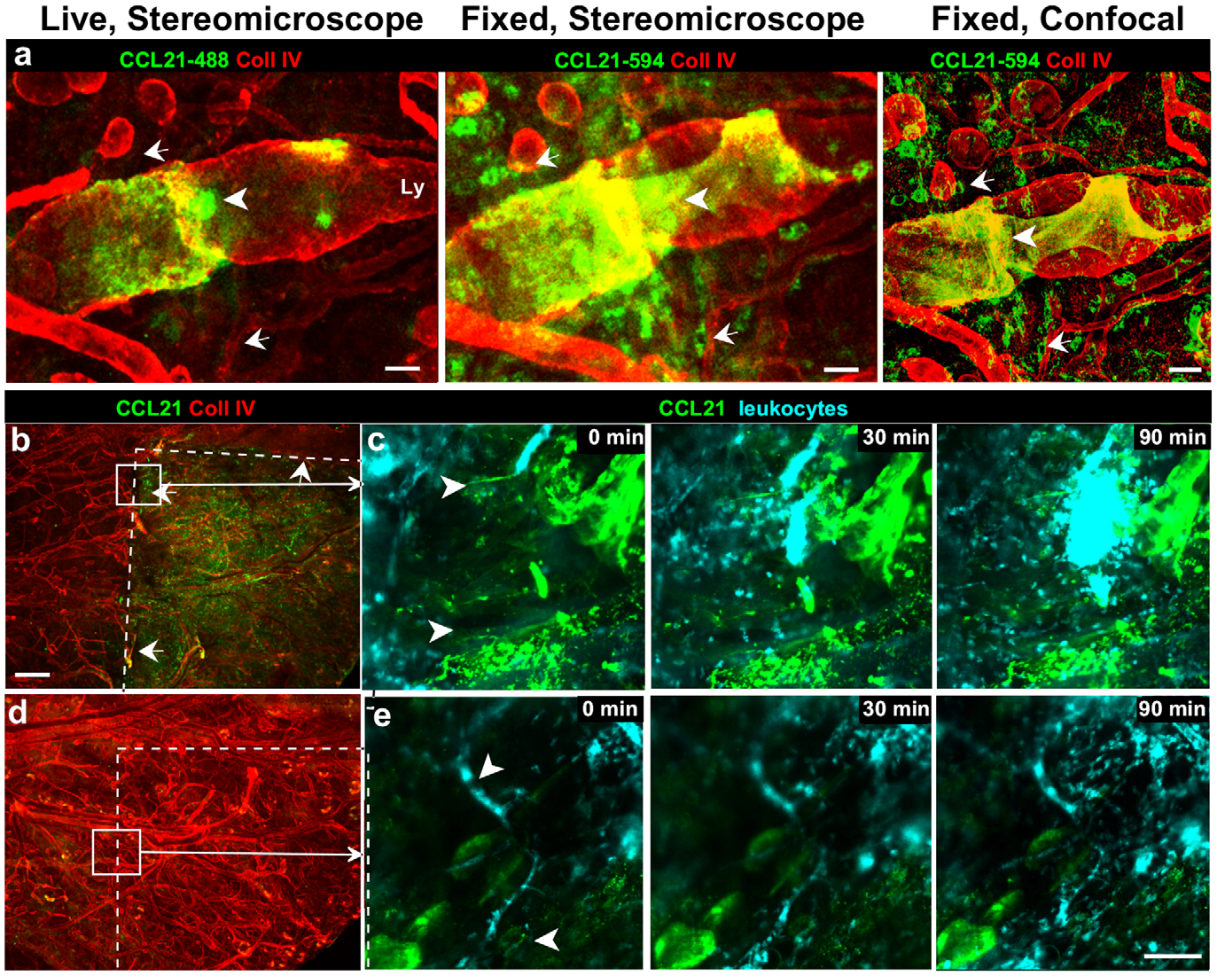
Intravital immunofluorescence identifies extracellular stores of CCL21. (**a**) Immunostaining in the live ear (left) reveals only extracellular CCL21, which is extensively deposited on fragments of basement membrane-rich collecting lymphatics. In contrast, after fixing and re-staining for CCL21 of the same tissue, wide-field epifluorescence stereomicroscopy (middle) and confocal microscopy (right), detected intracellular stores of CCL21. Arrowhead points to CCL21 deposit on collectors basal membrane that were detected with intravital IF; arrows point to example location where peri-nuclear (intracellular) CCL21 signal develops only after the same tissue is fixed and permeabilized before re-staining. (b–e) Anti-CCL21 antibody does not substantially block activity of CCL21 deposits. (b) Extrinsic mouse CCL21 (50 µg/ml) was applied topically on a small delineated portion of the exposed ear dermis, and was observed bound to extracellular matrix and cell surfaces, and was detected with an antibody against intrinsic CCL21. (c) Immunostaining with anti-CCL21 antibodies did not block signaling that drove the directional migration of bone marrow-derived EGFP leukocytes towards CCL21 that had been pre-adsorbed on the tissue matrix. (d) When exogenous CCL21 was applied to the ear dermis at only 1 µg/ml, CCL21 could not be detected in the exposed part of the extracellular matrix, and at this 50-fold lower concentration, directed migration of leukocytes towards the region was absent. Instead bone marrow-derived EGFP leukocytes migrated towards the direction of the intrinsic CCL21 deposits (e). Arrows in b and d point to the edge of the topically applied CCL21 zone. Arrowheads in c and e point the direction of cell movement. Detection reagents in a: collagen IV-streptavidin Alexa 647, CCL21-Alexa 488 (stereomicroscope), CCL21-Alexa 594 (confocal). In: b–e: collagen IV-streptavidin Alexa 647, CCL21-Alexa 488. Scale bars in a, 50 µm; b and d, 500 µm; c and e, 100 µm.

To confirm that our intravital IF staining for CCL21 was specific, after live-labeling we sacrificed the mouse, fixed and permeabilized the ear dermis, and re-probed it for CCL21 using a standard whole-mount preparation [26] (Figure 5a). In addition to the extracellular CCL21 patches that we had seen with the live staining (Figure 5a, left), extensive perinuclear localization in pre-collecting lymphatic endothelial cells were observed in the fixed tissue (Figure 5a, middle and right). This observation highlights another advantage of intravital IF: namely, that it labels only extracellular, matrix-bound chemokine deposits (which are relevant for chemotactic signaling) from biologically inactive intracellular stores, thus allowing the identification of potential entry gates for immune cells.

Finally, to determine whether immunostained CCL21 in live tissues interfere with its function, we incubated exogenous recombinant CCL21 on a well-defined portion of the exposed skin (Figure 5b), then immunostained for CCL21, and analyzed leukocyte migration towards this artificially formed zone of basement membrane-bound CCL21 (Figure 5c). Staining of exogenous, matrix bound CCL21 additionally served as a control for specificity of the antibody staining. We compared two doses of exogenous CCL21∶50 µg/ml vs. 1 µg/ml; the higher dose stained abundantly (Figure 5b) and chemoattracted blood vessel extravasation leukocytes (Figure 5c, Video S7) while deposition of the 50-fold lower dose of CCL21 was undetectable (Figure 5d) and did not attract migration of leukocytes. Instead, leukocytes entered the immunostained area of intrinsic CCL21, migrating against potential gradient of applied extrinsic chemokine. (Figure 5e and Video S8). These experiments also showed that immunolabeling of chemokine with antibodies interfering with their biological functions do not completely block preformed CCL21 gradients.

### Intravital Imaging of Leukocytes

**Figure 6:**
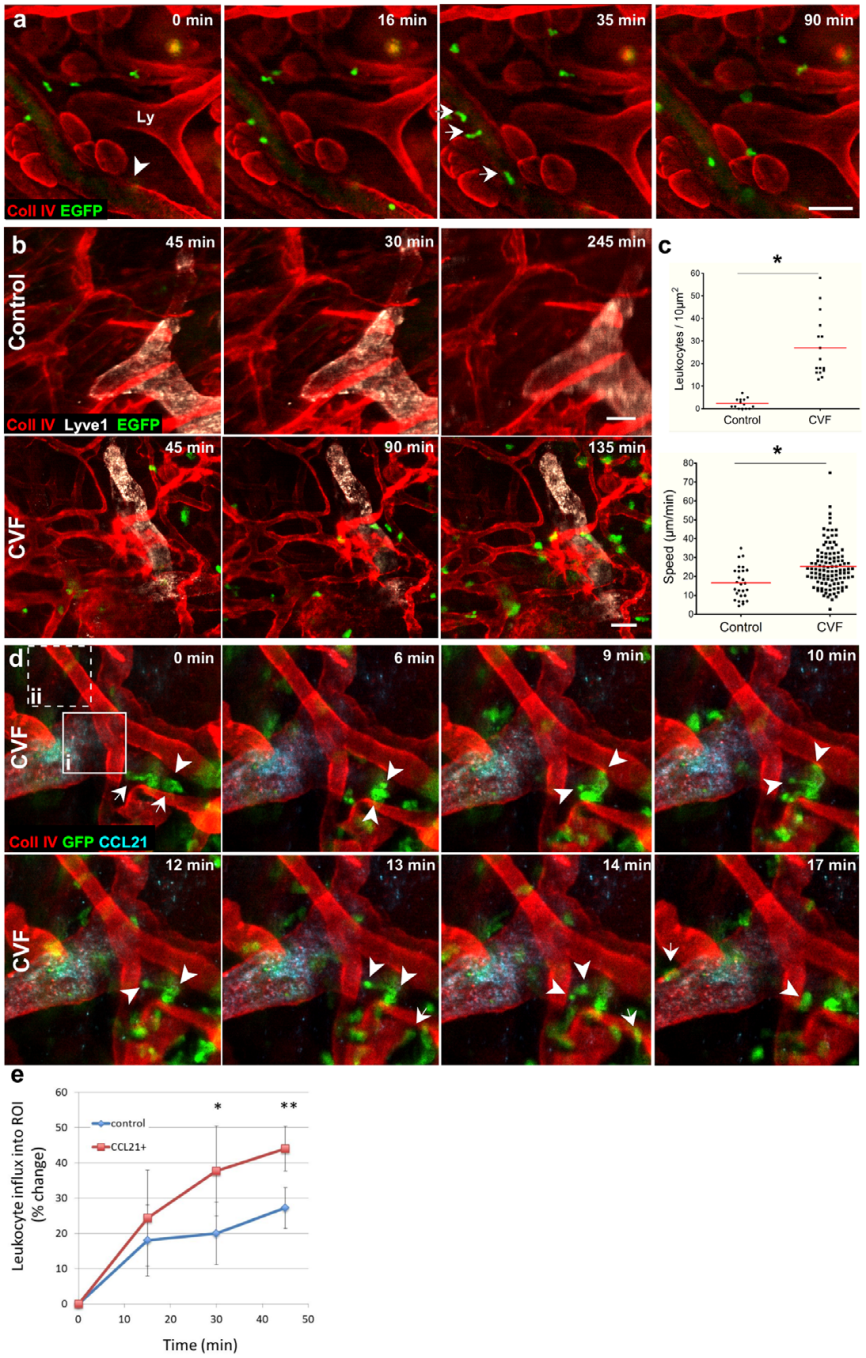
Leukocyte migration and extravasation events in anaphylatoxin-stimulated tissue can be imaged with intravital immunofluorescence (IF). (**a**) Sequential images of the ear dermis of a wild-type mouse, stained for collagen IV (red) and Lyve1 (white) and transfused with blood from an EGFP mouse. Tissue stimulated with cobra venom factor (CVF, an anaphylatoxin)-treated serum caused leukocytes (arrows) to roll within a venule (arrowhead), transmigrate across venules, and migrate within the tissue. (**b**) Sequential images show the extent to which stimulation with serum and CVF (anaphylatoxins, bottom row) drives massive extravasation of leukocytes from blood vessels, compared to that under control conditions (top row). (**c**) Quantification of tracked leukocytes does not only show 15 times increase in leukocytes extravasation after CVF treatment, but also that their migration speeds were significantly faster. (**d**) Sequential images from an EGFP-chimeric mouse showing number of leukocytes entering CCL21-zone with a single leukocyte potentially entering pre-lymphatic collecting vessels and then stopping at the basement membrane for 8 minutes (6 to 14 min), squeezing its cytoplasm at the level of the CCL21-positive pre-collecting lymphatic vessel (9 to 14 min), and subsequently gaining the rounded-cell morphology (17 min). Arrowheads point to front and tail of the potentially transmigrating cell. Arrows point to cells that are crawling over basement membrane of the collecting lymphatic vessel. Tissue was stimulated with anaphylatoxins from CVF treated serum. Squares in image at 0 min represent example regions of interests (ROIs) that were placed onto either a CCL21-positive zone (i) or in a control zone (ii, dashed square) and used to calculate the relative density of cells entering CCL21 and control zones. (**e**) Leukocytes preferentially migrated towards intrinsic CCL21 immunolabeled depots. Cross correlation intensity from 6 pairs of sequential images increases in CCL21-ROIs and it is significantly different from control-ROIs, with p-value declining from 0.13 (15 min), 0.03 (30 min), to 0.008 (45 min). Error bars represent standard deviation. In: b–e: collagen IV-streptavidin Alexa 647, CCL21-Alexa 488. A–c, blood from EGFP mouse was transfused to WT after the tissue was stained with antibodies as indicated; d and e, bone marrow from EGFP mouse was transplanted to WT mouse two months before the experiment. Detection reagents: collagen IV-streptavidin-Pacific Blue, CCL21-Alexa 594, white-Lyve1-Alexa 488. Scale bars, 100 µm.

Next, to examine leukocyte extravasation from venules, we performed intravital IF on mice that had received whole blood transfusions from an EGFP-mouse, which expresses *EGFP* under the ubiquitin promoter, making every cell (including erythrocytes) fluorescent. After collagen type IV staining, leukocyte extravasation was stimulated with anaphylatoxins from cobra venom factor (CVF)-activated mouse serum [12], [32], [33]. After that, a single post-capillary venule was imaged every 30 s for 1.5 h (Figure 6a and Video S9), during which time several leukocytes were observed extravasating from the venule after crawling along the basement membrane (Figure 6a).

The cumulative effects of the surgery and staining on tissue activation was assessed by comparing spontaneous vs. anaphylatoxin-induced leukocyte transmigration in the Lyve-1 and collagen IV-stained dermis for up to 12 h (Figure 6b). Importantly, we saw that the rate of leukocyte extravasation in unstimulated tissue was minimal (Figure 6b, top, Figure 6c and Video S10), while that in anaphylatoxin-stimulated dermis was enhanced 15-fold (Figure 6b bottom, Figure 6c and Video S11). Furthermore, anaphylatoxin-stimulated cells moved faster after extravasation than cells under resting conditions, with average speeds of 25±12 µm/min vs. 17±9 µm/min (mean±SD), respectively. This was consistent and within a range of previously reported migration speeds for neutrophils (30 µm/min) and monocytes (5 µm/min) [34], cells that most numerously extravasate from blood after stimulation with anaphylatoxins [35] (Figure 6c). Therefore, while the surgical separation of the ear and subsequent immunostaining of the tissue may induce an acute inflammatory response, this was minimal compared to that stimulated by activated complement.

Activated mouse neutrophils can be chemoattracted to lymphatic-derived CCL21, traffic to lymph nodes, and stimulate adaptive immune responses similar to migratory dermal DCs [36]. We investigated lymphatic interactions with extravasated granulocytes (the majority of which are neutrophils) and monocytes in anaphylatoxin-stimulated dermis stained for collagen IV and CCL21 and observed numerous EGFP^+^ leukocytes crawling along the CCL21-positive areas of collecting lymphatics. Because of the lack of vertical sectioning in the stereomicroscope, we could not definitively identify intravasation into lymphatic vessels, but potential intravasation events were associated with morphological and behavioral changes of the migrating cells. In contrast to cells crawling over basement membrane, which move continuously and with relatively little change in cell shape, some cells were observed to stop moving for several minutes at the margin of the vessels, deform dramatically as if squeezing through a vessel wall and then rounding up before moving again (Figure 6d and Video S12). Since such behaviors and shape changes are associated with transendothelial migration [37], these behavioral and phenotypic indicators might be used a hallmark of intravasation events into the lymphatic vessel.

We calculated the preferred directionality of cells migrating into control and CCL21 areas. Intensity of signals from six pairs of cross-correlated images, CCL21-negative and CCL21-positive region of interest (ROI) showed that extravasating leukocytes accumulate preferentially in CCL21 positive zones (Figure 6e). This indicated that chemokine naturally deposited on basement membranes can presumably form gradients to attract cells in the immunolabeled preparation of the exposed ear dermis.

**Figure 7:**
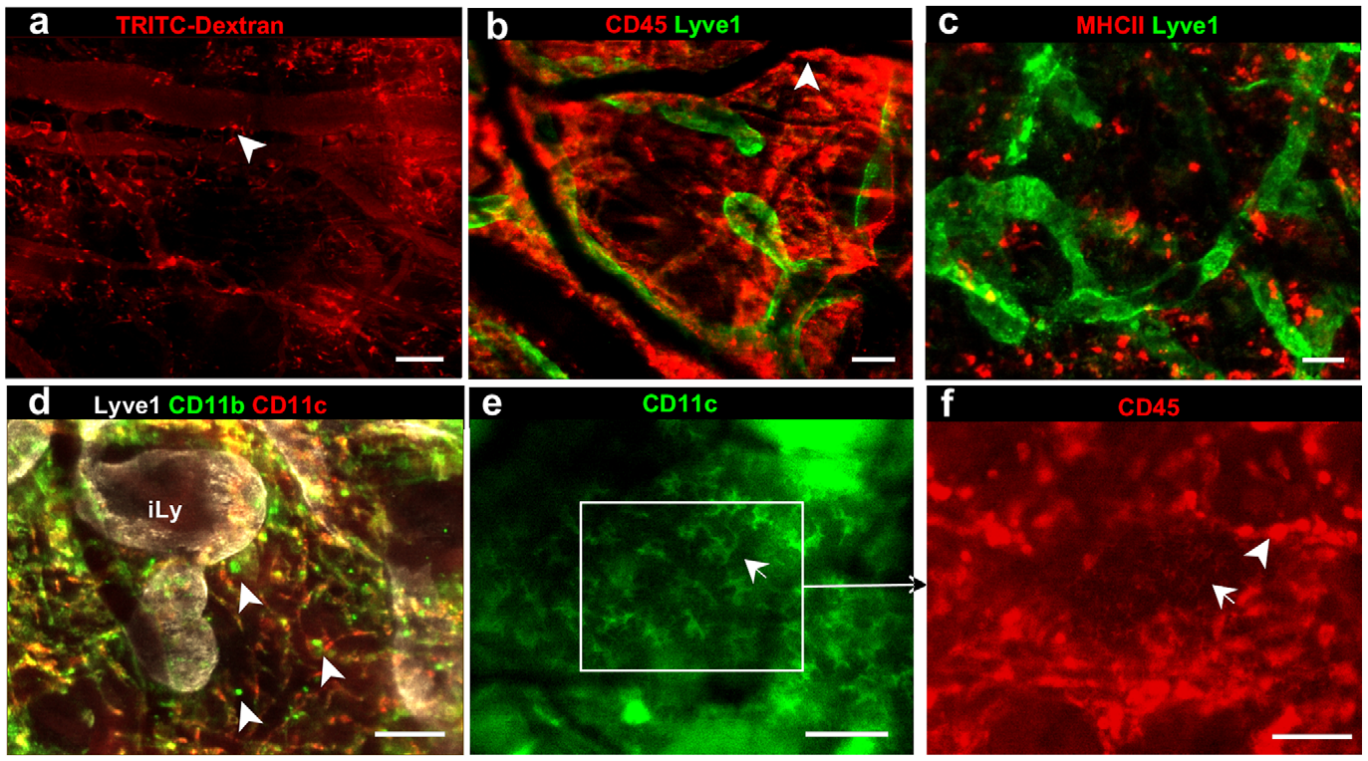
Immunostaining for various cell-surface markers differentiate between resident immune cell types. (**a**) 155 kDa TRITC-dextran, injected intradermally two months prior, is seen inside tissue-resident macrophages (arrowheads). (**b–g**) Differential identification of various leukocyte subsets as indicated: (**b**) all leukocytes express CD45, while (**c**) activated macrophages and dendritic cells express MHCII, (**d**) macrophages express CD11b (which can either be CD11c^+^, yellow, or CD11c^−^, green) and dermal dendritic cells (DDCs) express CD11c (red). (**e**) DDCs and Langerhans cells (identified by their characteristic morphology, arrow) express CD11c, but (**f**) Langerhans cells express lower levels of CD45 than other tissue resident immune cells. Because Langerhans cells reside in epidermis of the skin, they can be visualized with regular epifluorescence microscopy only in the areas of the skin free of macrophages. Detection reagents used: CD45-streptavidin Alexa 647, MHCII-Alexa 594, Lyve1-Alexa 488, CD11b-Alexa 647, CD11c-Alexa 594 (red, panel d) or CD11c-Pacific Blue (directly conjugated antibody, panel e). Scale bars, 50 µm.

We also tested numerous methods to visualize leukocyte subsets of the mononuclear phagocyte system (Figure 7), including perivascular macrophages that can be visualized after i.d. injections of TRITC-dextran, which they phagocytosed [12]. Similar staining patterns were obtained by labeling for macrophage and DCs subtypes using the pan-leukocyte marker CD45. MHCII+ macrophage and dendritic cell populations were distinguished with differential staining for CD11c and CD11b. Epidermal CD11c+ Langerhans cells could be identified by their characteristic dendrite morphology [17] and only in the areas where there were no dermal macrophages present in the first imaging plane that normally mask the presence of Langerhans cells.

### Heterogeneous Migratory Behaviors of Different Leukocyte Subsets in vivo

**Figure 8:**
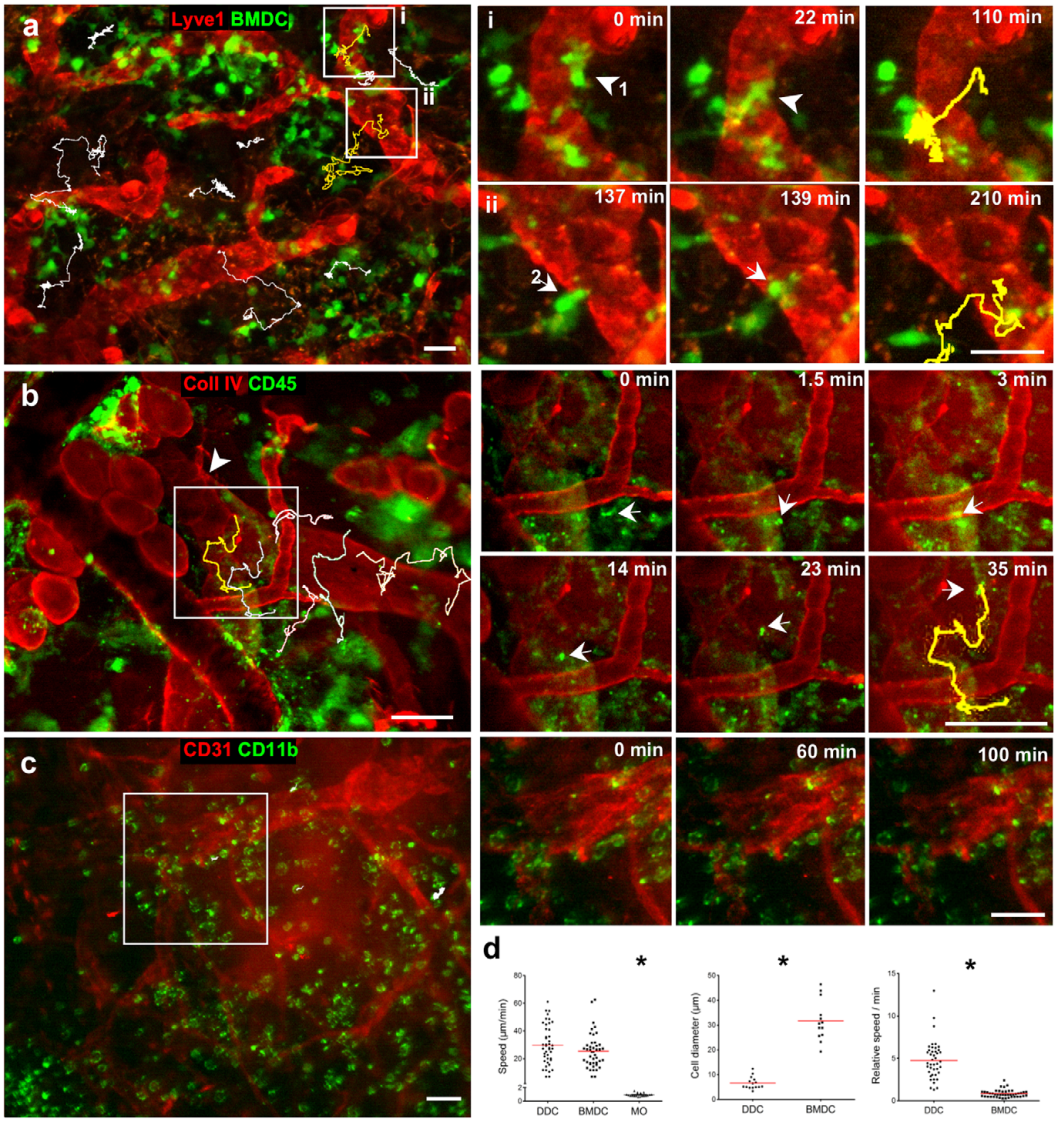
Intravital immunofluorescence reveals different migratory behavior between leukocyte subsets in the skin. (**a**) Bone marrow-derived dendritic cells (BMDCs), isolated from the EGFP mouse and matured with LPS, were overlaid onto the Lyve1-α-rabbit Alexa 488 (red) stained ear dermis, and after 30 min the tissue was imaged for 3 hours. BMDCs were tracked (example tracks shown in white); (i) and (ii) show sequential images of two indicated areas where BMDCs (arrows, with yellow tracks shown at right) were observed entering Lyve1^+^ initial lymphatic vessels. (**b**) After staining for collagen IV (streptavidin-Alexa Pacific Blue, red) and CD45 (Alexa 596, green), the ear skin was stimulated with CVF-treated serum (anaphylatoxins) and imaged. Left, arrowhead points to pericyte imprint, indicative of collecting lymphatic vessels. Migrating CD45^+^ cells, most likely dermal dendritic cells (DDCs), were tracked (white). Insets (i) and (ii) show DDCs (arrows) entering collecting lymphatic vessels, with their migratory tracks (yellow) shown at right. (**c**) *Ex vivo* isolated peritoneal macrophages were stimulated with LPS and stained for CD11b-Alexa 594 before overlaying onto PECAM-1-Alexa 488 stained dermis. After 30 min, the tissue was imaged for 2 hours. Inset area (white box, left; sequential images, right) highlights the slow speed of migration of these cells compared to the DCs. (**d**) Quantification of cell migration speeds in (a)–(c). BMDCs and DDCs migrated with roughly the same speeds while macrophages remained immotile; since BMDCs were 5 times larger than intrinsic DDCs, their relative speed of migration (speed divided by cell diameter) was 5 times slower (p<0.0001). Scale bars, 50 µm.

Many studies of DC migration involve the tracking of bone marrow-derived DCs (BMDCs), which after isolation, differentiation and activation, are placed on excised ear skin [16] or observed *in vitro* [19]. We compared the migratory behavior of EGFP-BMDCs placed on the live, immunostained ear tissue 30 min before imaging (Figure 8a and Video S13) with that of intrinsic CD45-labeled dermal DCs (DDCs) (Figure 8b and Video S14) and *ex vivo* labeled peritoneal macrophages (Figure 8c and Video S15).

In untreated tissue, majority of CD45 cells were immotile (Video S5), while anaphylatoxin stimulation induced a DDC migratory phenotype (Video S14). In contrast to DCs, LPS-stimulated murine peritoneal macrophages that had been *ex vivo* stained for CD11b, stimulated with LPS and overlaid on the ear (pre-stained for CD31) entered the tissue but remained immotile (Figure 8c and Video S15). Quantification of cell migratory tracks demonstrated that BMDCs and DDCs migrated with similar average speeds (Figure 8d). However, BMDCs were 5 times larger than intrinsic DDCs, thus the relative speed of BMDCs (speed/cell diameter) was significantly lower than that of DDCs (Figure 8d). In both cases we observed potential lymphatic intravasation events where cells appeared to enter into initial lymphatic vessels (BMDCs, Figure 8a) or pre-collecting lymphatic vessels (DDCs, Figure 8b) according to their morphological and behavioral changes as described above.

CD11c is a commonly-used marker for intrinsic DCs, but immunostaining for CD11c can interfere with DDC functions including their migration. We thus used the less specific DC surface receptor CD45 and CD11b to validate the results obtained from CD11c-stained DDCs, distinguished from sessile macrophages by their migratory behavior on time-lapse image series (Figure 8 and Video S14 and S15). Also, we do not expect large number of CD45^+^ granulocytes as number of infiltrating leukocytes was negligible during 30 min staining in unstimulated tissue (Figure 6b and Video S10).

## Discussion

The intravital imaging technique described here provides a low-cost, technically simple, and highly versatile means of intravital imaging that allows fluorescent cells, labeled either with reporter genes or intravital IF, to be observed in the context of immunostained extracellular matrix components and chemokine stores while simultaneously assessing blood and lymphatic vessel function. Our technique combines the flexibility of immunostaining with the physiological relevance of intravital imaging, where intact blood and lymphatic vessels maintain the natural currents of interstitial flows that affect chemokine gradients and therefore cell migration. Surgical exposure and subsequent immunostaining of the ear dermis were simple and quick procedures that could be completed in less than one hour. The nearly 2-dimensionality of the ear dermis allowed a wide-field fluorescence stereomicroscope, equipped with an automatic stage, to be used, which could simultaneously monitor up to 20 fields magnified at 320 times at 0.95 µm resolution in a single ear. Ear immobilization and sustained delivery of antioxidants allowed live imaging for at least 12 h without noticeable adverse effects on the tissue, behavior of imaged cells, or well-being of the mouse.

Using this method we identified the existence of highly concentrated basement membrane-bound CCL21 chemokine deposits, distinguishable from biologically inactive intracellular stores. By imaging leukocyte migration behaviors in living tissues, our observations suggest that in addition to known sites of entry into the initial lymphatics [38], leukocytes might also intravasate into pre-collecting lymphatic vessels, where discrete patches of extracellular CCL21 can be found.

Although immunostaining in live tissue has been performed previously, e.g. to image circulating platelets [7] or tumor-associated myeloid cells [12], its general use is limited by humoral-mediated immunotoxicity and phototoxicity. The method described here addresses these challenges, thereby allowing the use of amplification steps to increase sensitivity and decrease staining background. On the other hand, this method has obvious limitations when using antibodies that interfere with function, and thus is most appropriate for markers that are not involved in the investigated events. Nevertheless, the relevance of the results should be confirmed in independent experiments that use different cell or structure antigens, e.g. CD11c, CD11b, or MHCII on DDCs or different basement membrane protein like collagen IV, perlecan or laminin. Other limitations of our system include using immunocomplexes to block F_c_γ receptors, inhibiting initial micro-hemorrhages from injured capillaries with aprotinin, and using the antioxidant ascorbate, which may adversely affect the tissue (but not intracellular) redox balance. Treatment with immunocomplexes and pro-coagulants can stimulate the production of inflammatory cytokines by endothelial cells and leukocytes and activate other tissue stroma cells [25]. It is possible to reduce immunocomplex-dependent tissue activation by using fluorophore-labeled secondary F(ab)_2_ or biotinylated primary antibodies with streptavidin-fluorophore detection; however, this will only lessen the immunotoxicity, as ligated primary antibodies can also activate F_c_-dependent biological functions. Nonetheless, we found that the activation of immunolabeled tissue was low with minimal granulocyte or monocyte extravasation (Figure 6b). Wide-field epifluorescence microscopy does not allow us to determine whether a cell is crossing an endothelial barrier in a definitive manner. Such events can only be confirmed with co-localization techniques using confocal sectioning. However, in wide-field imaging these events may be indirectly identified according to behavioral and morphological changes of intravasating cells (Video S12, Figure 6d and [15]). This could lead to potential identification of false-positive events; however, the simultaneous collection of large datasets may compensate for that disadvantage by increasing the probability of capturing the true incidents.

Because the signal strength from indirect immunofluorescence was above the background fluorescence in the visible light spectrum, we did not observe any differences in skin autofluorescence between BALB/c, C3H and C57 mice (not shown). Hence, different genetic mouse strains expressing fluorescent reporter proteins, e.g. the ProxTom and the Vegfr3(EGFPLuc) [39] lymphatic reporter mouse or R26R-Confetti mouse [40] can additionally enrich the array of available imaging options. Our technique can readily incorporate classical cell labeling methods as we demonstrated by imaging the interactions of transplanted EGFP-expressing DCs or granulocytes and extracellular matrix staining for collagen IV or Lyve1 (Figure 6 and 8). Hence the methods presented here could be combined, for example, with the widely used dorsal skinfold chamber model [41] or to visualize tumor microenvironment [42]. Furthermore, with appropriate modifications, intravital IF can be used for a variety of alternative applications, including imaging skin-infecting parasites [43], mechanisms of angio- and lymphangiogenesis [44] and skin transplant immunity [45].

## Supporting Information

Figure S1 **Separating the skin flaps resulted in minimal trauma in the dorsal dermis.** Exposition of the ear dorsal dermis from ventral skin and cartilage. (**a**) The ears and surrounding cranium skin were depilated at least 3 days before the surgery. (**b**) The ventral ear skin and (**c**) underlying cartilage were cut along the antihelix. (**d**) The edges of the skin were cut, and (**e–f**) the ventral skin with underlying cartilage was pulled towards the tip of the ear. (**g**) For subsequent antibody staining and imaging, the intact eminea concha of the mouse ear was attached to a glass slide with surgical glue; this glass slide was fixed to the microscope base. During staining, the exposed dermis was placed on Parafilm, which was replaced with a coverslip before imaging. A needle, connected through a peristaltic pump to a reservoir of ascorbate-Ringer solution to bathe the ear throughout the imaging session, was placed under the coverslip and the mouse was kept under 1.5% isofluorane for up to 12 hours of imaging.
(TIFF)

Figure S2 **Efficient intravital immunofluorescence requires intact lymphatic drainage and the use of secondary antibodies for signal amplification.** **(a)** Comparison of signal-to-background ratio between (left) directly stained lymphatic capillaries with Alexa 488-labeled rabbit anti-mouse Lyve1 antibody, and (right) the same area after subsequent incubation with Alexa-488-labelled goat α-rabbit IgG. **(b)** Comparison of immunostaining efficiency in a tissue area with functional lymphatic drainage (top) and an adjacent area where the functional lymphatic drainage was interrupted by a laceration at the base of the ear (bottom). Scale bars in a, 500 µm; b, 200 µm.
(TIFF)

Figure S3 **Intravital immunolabeling was dependent on intradermal flows and could distinguish between various compartments of the microvasculature. (a)** Due to local convective transport within the dermis, more anti-PECAM-1 antibody (red) was directed to lymphatic vessels and non-perfused blood vessels than to functional blood vessels where fluid convection is in the opposite direction. This leads to stronger staining of injured blood vessels with blocked flow. The ear dermis was stained for PECAM-1-Alexa 594 (red) and collagen IV-Alexa 647 (blue), and an i.v. injection of 2000 kDa FITC-dextran (green) allowed a clear distinction between perfused blood vessels. “Flow”, correlated with weak PECAM-1 staining and non-functional blood vessels. “No flow”, which correlated with stronger PECAM-1 staining. Lymphatic vessels (Ly) could be distinguished by their specific morphology. **(b)** Lyve1 staining (green) on initial lymphatics (iLy) was inconsistent and discontinuous, while collagen IV staining (red) delineated the entirety of the lymphatic capillary network. **(c)** In addition to stronger collagen IV staining, pre-collecting lymphatics (Ly) could be distinguished from initial lymphatic capillaries by their differential expression of podoplanin and Lyve1, respectively. **(d)** Lyve1-positive and collagen IV^dim^ initial lymphatic vessels (in focus,eft) were located closer to the epidermis then the Lyve1-negative collecting vessels (in focus right). Arrows indicate the direction of lymph flow as deduced from valve orientation. **(e)** CCL21 (green) was mostly observed on collecting lymphatic vessels, and stained more weakly on initial lymphatic capillaries (arrow). Scale bars, 100 µm.
(TIFF)

Video S1 **Demonstration of surgical procedure for separating the ventral skin and cartilage from underlaying dorsal skin.** For this demonstration, the mouse was anesthetized with 50 mg/kg ketamine and 10 mg/kg xylazine. Duration: 3 min 20 s.
(AVI)

Video S2 **Bright-field microscopy showing a fully intact blood circulation in the dorsal ear dermis after surgical removal of the ventral dermis and cartilage.** Duration: 1 min.
(AVI)

Video S3 **Immunofluorescence imaging, (5 frames/s) of collagen IV (green)-stained dermis that was i.v. injected with 100 µl of 20 mg/ml 155 kDa TRITC-dextran (red).** Duration: 10 min.
(AVI)

Video S4 **Functional lymphatic capillaries (arrows) collecting i.v. infused FITC-dextran (155 kDa) after leakage from blood vessels immediately after surgery.** Duration: 9 min.
(AVI)

Video S5 **Migration of DDCs and tissue-probing sessile macrophages (CD45-alexa 594, red) in Lyve1-Alexa488 (green) stained tissue were not apparently altered over 12** **h imaging, indicating lack of substantial phototoxicity, immunotoxicity or oxygen deprivation during this time.** Images were taken every 35 seconds in each channel. Duration: 12 hours.
(AVI)

Video S6 **Lymphatic staining occurs within minutes after the application of secondary (donkey anti-rabbit Alexa 488, green) antibody on Lyve1-labeled tissue.** Imaging started 2 minu after application of the secondary antibody. Duration: 18 min.
(AVI)

Video S7 **Anaphylatoxin stimulated leukocytes migrate towards matrix zone pre-incubated with 50 ng/ml extrinsic, recombinant CCL21.** Duration: 174 min.
(AVI)

Video S8 **Anaphylatoxin stimulated leukocytes migrate towards intrinsic deposits of CCL21 located at the lymphatic collector and not toward the tissue zone pre-incubated with 1 ng/ml extrinsic CCL21.** Duration: 152 min.
(AVI)

Video S9 **Transfused EGFP+ leukocytes (green) extravasate across a collagen type IV-labeled venule (red) after the tissue was stimulated with anaphylatoxin-activated serum.** Duration: 90 min.
(AVI)

Video S10 **Without anaphylatoxin stimulation, very few i.v. transfused EGFP+ leukocytes (green) extravasate from blood venules after surgery and immunostaining.** Secondary reagents used: Lyve1–Alexa 594 (white), collagen IV–streptavidin-Alexa 647 (red). 25% of leukocytes were EGFP-labeled. Duration: 285 min.
(AVI)

Video S11 **After anaphylatoxin stimulation, a massive extravasation of i.v. transfused EGFP+ leukocytes (green) were observed.** Secondary reagents used: Lyve1–Alexa 594 (white), collagen IV–streptavidin Alexa 647 (red). 25% of leukocytes were EGFP-labeled. Duration: 115 min.
(AVI)

Video S12 **In anaphylatoxin-stimulated tissue, EGFP+ leukocytes (green) were observed crawling towards and onto a pre-collecting lymphatic vessel near a highly concentrated CCL21-deposit.** A possible intravasation event (arrow) was hypothesized based on cell behavioral and morphological changes such as arrested cell movement and drastic shape changes in the cell. Furthermore, numerous invading cells were observed to crawl over lymphatic basement membrane without cell arrest. Secondary reagents used: CCL21–Alexa 594 (cyan), collagen IV–streptavidin Alexa 647 (red). Duration: 46 min.
(AVI)

Video S13 **EGFP^+^ LPS-matured dendritic cells, extracted from bone marrow, were observed intravasated (arrows) into Lyve1-stained lymphatic vessels (red) matured with LPS for 24 hours were overlaid for 30 minutes on the Lyve1-stained dermis. Secondary reagents used: Lyve1-Alexa 594 (red).** Duration: 5.2 h.
(AVI)

Video S14 **Tissue basement membrane and intrinsic DDCs were stained for collagen IV and CD45, respectively, and then stimulated with anaphylatoxin from cobra venom factor-activated serum.** While the majority of CD45+ cells remained immobile, several exhibited migratory behavior and entered (arrow) pre-collecting lymphatic as deduced from their shape change during and after intravasation. Secondary reagents used: collagen type IV-streptavidin-Pacific Blue (white). Duration: 90 min.
(AVI)

Video S15 **Peritoneal macrophages** ***in vitro*** **stimulated for 24 hours with 1 **µ**g/ml of LPS and labeled for CD11b (green).** Cells entered and spread in the dermis but did not display any migratory behavior. Blood and lymphatic vessels were stained for PECAM-1-Alexa 488 (red). Duration: 110 min.
(AVI)
