# Supplementary figures and images for: Intravital Immunofluorescence for Visualizing the Microcirculatory and Immune Microenvironments in the Mouse Ear Dermis
Source: PLoS One. 2013 Feb 25;8(2):e57135. doi: 10.1371/journal.pone.0057135 (PMC3581585; doi:10.1371/journal.pone.0057135)

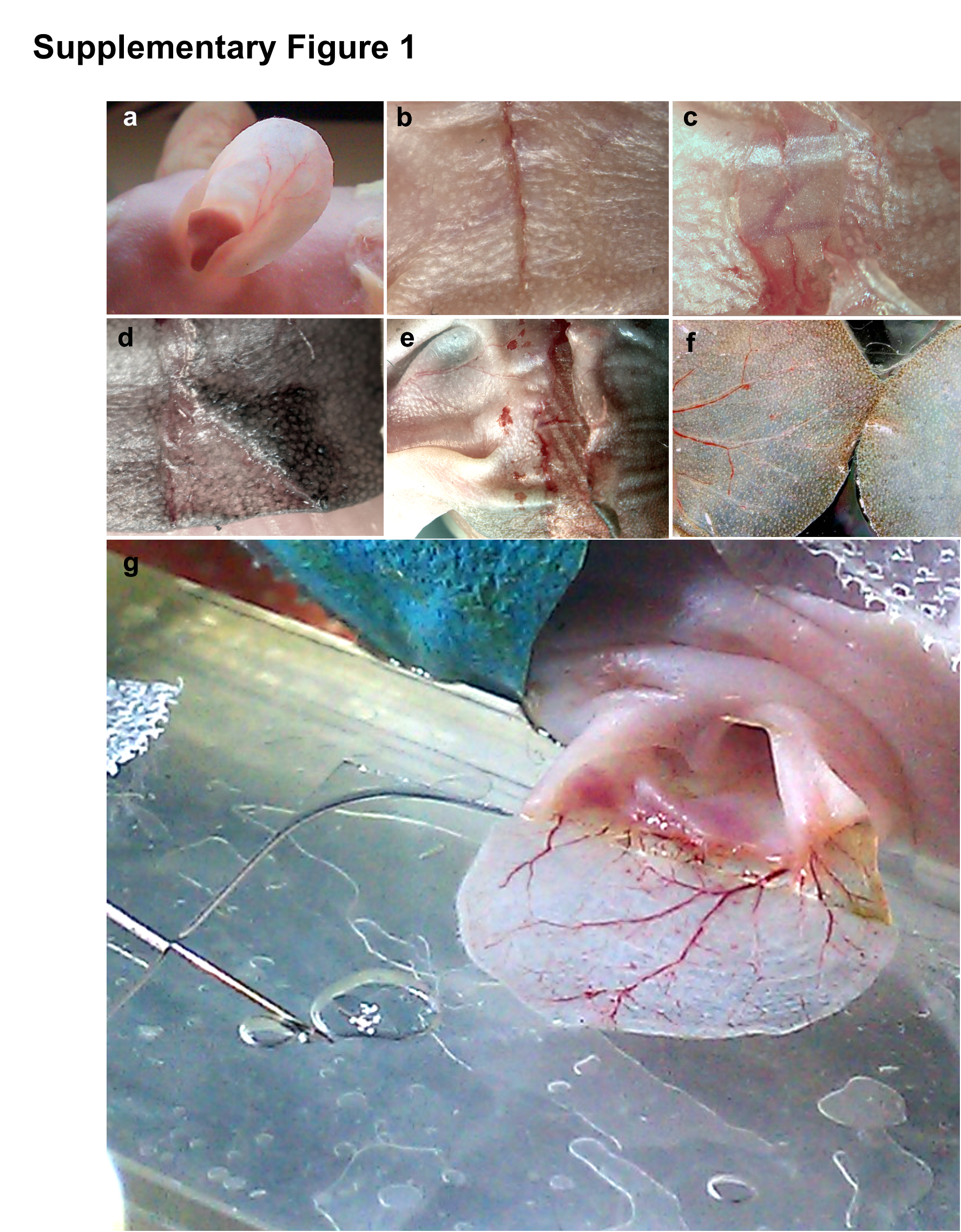

Supplement: Figure S1 — Separating the skin flaps resulted in minimal trauma in the dorsal dermis. Exposition of the ear dorsal dermis from ventral skin and cartilage. (a) The ears and surrounding cranium skin were depilated at least 3 days before the surgery. (b) The ventral ear skin and (c) underlying cartilage were cut along the antihelix. (d) The edges of the skin were cut, and (e–f) the ventral skin with underlying cartilage was pulled towards the tip of the ear. (g) For subsequent antibody staining and imaging, the intact eminea concha of the mouse ear was attached to a glass slide with surgical glue; this glass slide was fixed to the microscope base. During staining, the exposed dermis was placed on Parafilm, which was replaced with a coverslip before imaging. A needle, connected through a peristaltic pump to a reservoir of ascorbate-Ringer solution to bathe the ear throughout the imaging session, was placed under the coverslip and the mouse was kept under 1.5% isofluorane for up to 12 hours of imaging. (TIFF) [file pone.0057135.s001.tiff]

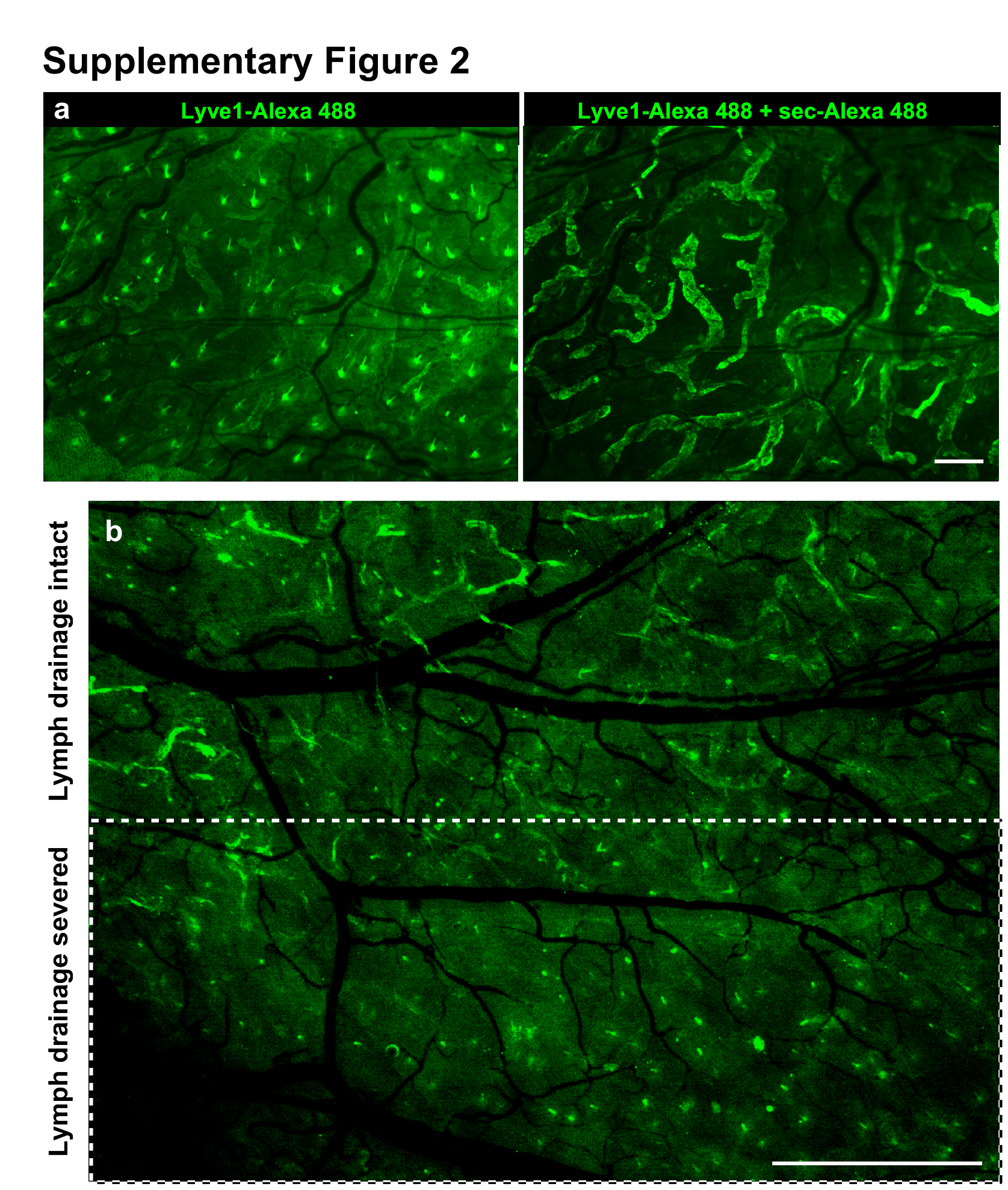

Supplement: Figure S2 — Efficient intravital immunofluorescence requires intact lymphatic drainage and the use of secondary antibodies for signal amplification. (a) Comparison of signal-to-background ratio between (left) directly stained lymphatic capillaries with Alexa 488-labeled rabbit anti-mouse Lyve1 antibody, and (right) the same area after subsequent incubation with Alexa-488-labelled goat α-rabbit IgG. (b) Comparison of immunostaining efficiency in a tissue area with functional lymphatic drainage (top) and an adjacent area where the functional lymphatic drainage was interrupted by a laceration at the base of the ear (bottom). Scale bars in a, 500 µm; b, 200 µm. (TIFF) [file pone.0057135.s002.tiff]

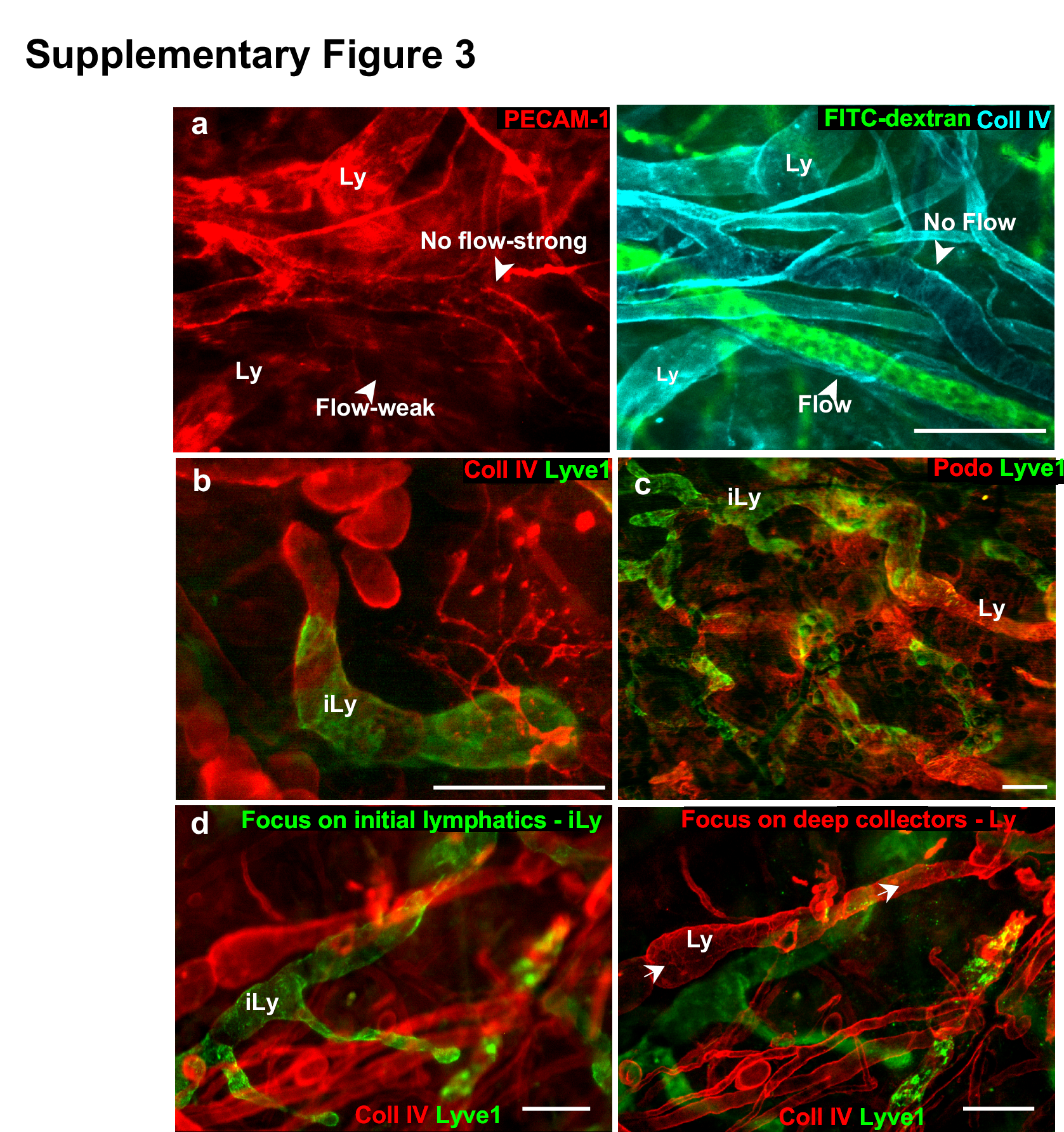

Supplement: Figure S3 — Intravital immunolabeling was dependent on intradermal flows and could distinguish between various compartments of the microvasculature. (a) Due to local convective transport within the dermis, more anti-PECAM-1 antibody (red) was directed to lymphatic vessels and non-perfused blood vessels than to functional blood vessels where fluid convection is in the opposite direction. This leads to stronger staining of injured blood vessels with blocked flow. The ear dermis was stained for PECAM-1-Alexa 594 (red) and collagen IV-Alexa 647 (blue), and an i.v. injection of 2000 kDa FITC-dextran (green) allowed a clear distinction between perfused blood vessels. “Flow”, correlated with weak PECAM-1 staining and non-functional blood vessels. “No flow”, which correlated with stronger PECAM-1 staining. Lymphatic vessels (Ly) could be distinguished by their specific morphology. (b) Lyve1 staining (green) on initial lymphatics (iLy) was inconsistent and discontinuous, while collagen IV staining (red) delineated the entirety of the lymphatic capillary network. (c) In addition to stronger collagen IV staining, pre-collecting lymphatics (Ly) could be distinguished from initial lymphatic capillaries by their differential expression of podoplanin and Lyve1, respectively. (d) Lyve1-positive and collagen IVdim initial lymphatic vessels (in focus,eft) were located closer to the epidermis then the Lyve1-negative collecting vessels (in focus right). Arrows indicate the direction of lymph flow as deduced from valve orientation. (e) CCL21 (green) was mostly observed on collecting lymphatic vessels, and stained more weakly on initial lymphatic capillaries (arrow). Scale bars, 100 µm. (TIFF) [file pone.0057135.s003.tiff]
